# Role of microRNA-34a in blood–brain barrier permeability and mitochondrial function in ischemic stroke

**DOI:** 10.3389/fncel.2023.1278334

**Published:** 2023-10-19

**Authors:** Cole T. Payne, Sidra Tabassum, Silin Wu, Heng Hu, Aaron M. Gusdon, Huimahn A. Choi, Xuefang S. Ren

**Affiliations:** Division of Neurocritical Care, Department of Neurosurgery, McGovern School of Medicine, University of Texas Health Science Center, Houston, TX, United States

**Keywords:** miR-34a, ischemic stroke, mitochondria, BBB permeability, microvascular endothelial cells

## Abstract

Over the past decade, there has been an uptick in the number of studies conducting research on the role of microRNA (miRNA) molecules in stroke. Among these molecules, miR-34a has emerged as a significant player, as its levels have been observed to exhibit a substantial rise following ischemic events. Elevated levels of miR-34a have been found to have multiple effects, including the modulation of inflammatory molecules involved in the post-stroke recovery process, as well as negative effects on the blood–brain barrier (BBB) permeability. Interestingly, the increase of miR-34a appears to increase BBB permeability post stroke, through the negative effect on mitochondrial function. The strength of mitochondrial function is crucial for limiting para-cellular permeability and maintaining the structural integrity of the BBB. Furthermore, the activation of ischemic repair mechanisms and the reduction of ischemic event damage depend on healthy mitochondrial activity. This review aims to emphasize the involvement of miR-34a in ischemic stroke, specifically its interaction with mitochondrial genes in cerebrovascular endothelial cells, the effect on mitochondrial function, and lastly its regulatory role in BBB permeability. A comprehensive understanding of the role of miR-34a in maintaining BBB integrity and its contribution to the pathogenesis of stroke holds significant value in establishing a foundation for the development of future therapeutics and diagnostic markers.

## 1. Introduction

Stroke is the second leading cause of mortality worldwide and the fifth leading cause in the United States (George et al., 2017). In 2019, there were 101.5 million recorded cases worldwide, with 77.2 million classified as acute ischemic strokes (Virani et al., 2021). Ischemic strokes were responsible for nearly 87% of all stroke cases in the United States (Hasan et al., 2021). Ischemic stroke is a neurological disorder characterized by cessation of blood flow to the brain as a result of clot formation in cerebral blood vessels. This blockage results in the death of neurons due to insufficient oxygen and nutrient supply. Due to the brain being the most metabolically demanding organ in the human body, loss of blood flow to the brain causes significant cognitive impairment, global metabolic process issues, and potentially death. Key events leading to stroke pathology include inflammation, cytokine-mediated toxicity, complement activation, tissue swelling, impairment of the blood–brain barrier (BBB), and free radical toxicity (Petraglia et al., 1987; Kuriakose and Xiao, 2020).

Ischemic stroke is closely associated with the disruption the BBB. Loss of the integrity of this barrier is responsible for neuronal tissue damage from influx of cytotoxic substances, brain edema, and oxidative damage. With a disrupted BBB, exogenous material is able to flood the brain via transcellular and paracellular routes. Due to the brains high rate of oxidative metabolism and elevated levels of polyunsaturated lipids, the brain is relatively vulnerable to oxidative damage (Kobeissy, 2015). Oxidative damage in stroke is multifaceted, involving the formation of reactive oxygen species (e.g., superoxide radicals) and increased production of nitric oxide following the initial BBB permeability increase. This damage persists even during reperfusion due to continued reactive oxygen species (ROS) generation. Extensive research has been dedicated to understanding BBB permeability in stroke outcomes, as it plays a significant role in stroke pathology. Hence, to investigate the BBB mechanisms in stroke is of critical importance since the post-ischemic increase in BBB permeability is what causes much of the damage associated with stroke.

Over the years, significant progress has been made in understanding the intricate relationship between stroke and the damage to the blood–brain barrier (BBB) (Geng et al., 2013). Stroke, particularly ischemic stroke, occurs when blood supply to a part of the brain is interrupted, leading to a shortage of oxygen and nutrients, which triggers a series of complex events. Oxidative damage is a multifaceted process in stroke (Kobeissy, 2015). It involves the generation of ROS like superoxide radicals, which cause oxidative stress and contribute to cellular injury (Niatsetskaya et al., 2012; Geng et al., 2013). The initial increase in BBB permeability triggers the release of nitric oxide, a signaling molecule, which further intensifies the oxidative stress. This oxidative insult leads to neuronal cell death, inflammation, and contributes to the expansion of brain injury during and after the stroke event (Nash and Ahmed, 2015). The ongoing research on BBB disruption in stroke is shedding light on novel therapeutic avenues. Strategies aimed at protecting and repairing the BBB, reducing oxidative stress, and modulating inflammation are being explored. Researchers are investigating various approaches, such as targeting specific molecular pathways involved in BBB integrity and developing innovative drug delivery methods that can selectively reach the brain. Furthermore, advancements in neuroimaging techniques have allowed for better visualization and understanding of BBB disruption in stroke patients (Kakkar et al., 2021; Wlodarczyk et al., 2022). Techniques like contrast-enhanced magnetic resonance imaging (MRI) and computed tomography (CT) scans provide insights into the extent of BBB damage, aiding in diagnosis and treatment planning (Kakkar et al., 2021; Lim et al., 2021).

The progress in stroke treatment has witnessed remarkable developments, transformed the landscape of care and improving outcomes for patients. While stroke remains a significant health challenge, particularly due to its potential for severe disability and mortality, advancements in various aspects of treatment have revolutionized the way strokes are managed. Despite many years of research, tissue plasminogen activator (tPA) remains the only FDA-approved therapy for stroke. This medicine works by dissolving clots in the brain, thereby mitigating the degree of oxygen deprivation during an ischemic event (Liu et al., 2018). Despite this, tPA is only effective in large artery occlusions and has a narrow window of effectiveness, making it time sensitive. Furthermore, its therapeutic index is relatively low due to the associated risks of side effects, such as exacerbation of brain edema and hemorrhage (Zhao et al., 2017). Treatment modalities during the second injury phase of stroke, which occurs within hours and days following the ischemic event, remain debatable and subject to clinical considerations at individual trauma centers (Ghajar, 2000; Shlosberg et al., 2010). More recently mechanical removal of brain clots combined with tPA thrombolysis is becoming a more common approach to treating stroke (Sacco et al., 2007; Barreto, 2011). This groundbreaking procedure involves physically removing the clot causing the stroke (Barreto, 2011). It is highly effective for large vessel occlusions and has demonstrated superior outcomes when combined with tPA treatment. Mechanical thrombectomy is often performed using minimally invasive techniques, such as threading a catheter through blood vessels to the site of the clot. This procedure has extended the treatment window significantly, offering hope to patients who might have been previously ineligible for intervention (Sacco et al., 2007). Nonetheless, further research is necessary to develop viable, and safe treatments for individuals that have experiences an ischemic event.

Given the substantial body of research demonstrating that many of the detrimental effects of stroke are caused by increased BBB permeability, the BBB is being the focus of many labs as a source of future treatments and medicines. Numerous studies on this process points to the role microRNA (miR/miRNAs) in the loss of BBB integrity (Ma et al., 2020; Wang et al., 2021). MiRNAs are small endogenous non-coding RNA molecules (20–25 nucleotides) that regulate gene expression by binding to a 3′ untranslated region (UTR) on gene transcripts (Li et al., 2021). Studies have previously found associations between the levels of specific miRNA and disease processes, including stroke. This review is focused on one specific miRNA, miR-34a which is known to be associated with BBB disruption and the pathophysiology of the stroke (Ren et al., 2019; Hone et al., 2020). While miR-34a is being reviewed in this paper, a more comprehensive understanding of association between this miRNA and stroke is required because the clinical significance of these molecules due to their potential as diagnostic markers and therapeutic targets.

## 2. The miRNAs

**Figure 1:**
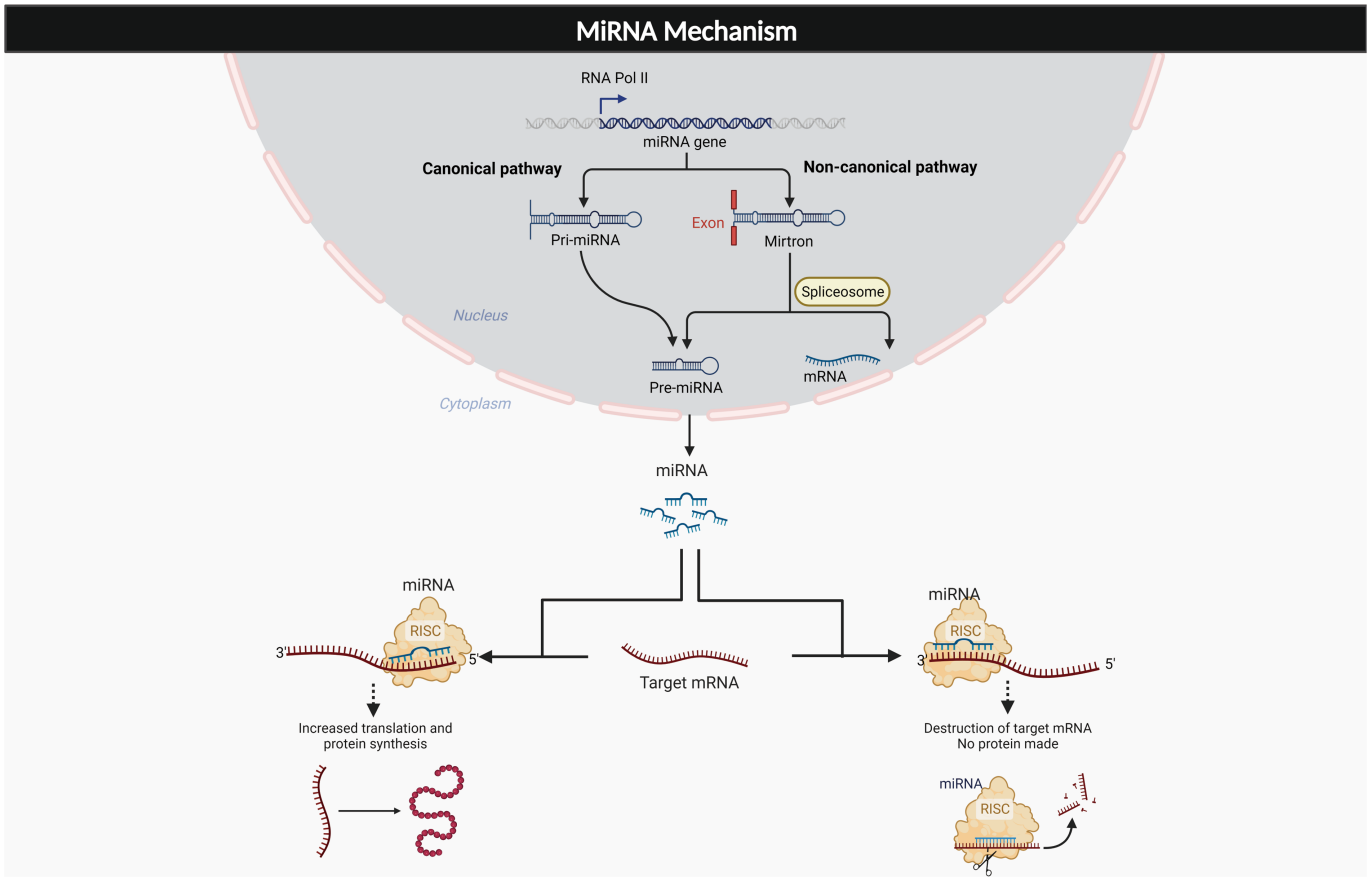
Generation and effects of miRNAs. The miRNAs are the complex molecule that is generated from two different pathways, canonical, and non-canonical pathway. The miRNA exerts multiple effects on target mRNA, dependent on its binding to 3’UTR, or 5’UTR.

MicroRNAs (miRNAs) are important regulators of gene expression that are found throughout the body. MiRNAs usually bind to the 3′ untranslated region of target mRNAs to suppress translation, and mRNA degradation. These molecules also have been found to bind to the 5’ UTR as well as to upregulate the translation process (O'Brien et al., 2018; Li et al., 2021). The generation of these molecules is described in two mechanisms which are canonical and non-canonical pathways. The canonical pathway of miRNA generation involves pri-miRNA that is processed within the nucleus and exported as a pre-miRNA duplex which is bound to miRNA- induced silencing complex (miRISC). The noncanonical pathway involves small hairpin RNA (shRNA) that are cleaved and exported to the cytoplasm where they are further processed and cleaved to miRISC. Both pathways lead to a functional miRISC complex, which binds to target miRNAs to induce translational inhibition (Hayder et al., 2018; O'Brien et al., 2018). As such, the interaction of miRNA and its target genes are very complex, resulting in a single miRNA having numerous potential effects throughout different portions of the body. These molecules have been estimated to control about 60% of protein coding genes in the human genome (Friedman et al., 2009) and an established function of these molecules has already been revealed through their role in cell differentiation, development and a variety of different metabolic processes (Barthels and Das, 2020). As Figure 1 depicts, the complexity of these molecules varies depending on where they bind to target mRNA, as well as the pathway in which they are generated, with the end result being a highly modifiable molecule. Due to the significant therapeutic potential and extensive involvement these molecules in a variety of disease processes, miRNA therapeutics is a major area of focus for many labs. Established relationships have been found with osteoporosis, diabetic neuropathy, cancer, Alzheimer’s, diabetic lymphoma, Parkinson’s, and stroke (Chakraborty et al., 2021). Table 1 depicts some of the most common pathologies involving miR-34a.

**Table 1:** Summary of diseases associated with miR-34a.

Disease	Mechanism involving miR-34a	Significance	References
Cancer	miR-34a is closely related to p53, and suspected to act as a tumor suppressor in many different types of cancer including breast cancer, thyroid cancer, etc.	MiR-34a has frequent decreased expression in cancer. Due to its downregulation in various cancers, miR-34a is considered a potential microRNA therapeutic candidate in cancer treatment.	Kalfert et al. (2020)
Diabetes	miR-34a directly regulates SIRT1, which is a molecule that regulates insulin release in the beta pancreatic cells that are damaged in diabetes.	Upregulation of miR-34a found in the diabetic heart and in the circulation from an early stage of the disease. SIRT-1 inhibition by miR-34a increases insulin secretion and adds oxidative stress to cells in diabetic heart.	Fomison-Nurse et al. (2018), Kitada and Koya (2013)
Hypertension	miR-34a levels increase in peripheral blood of patients with hypertension.	Upregulated miR-34a may promote vascular endothelial injury by affecting TIGF2 molecules.	Hua et al. (2021)
Myocardial infarction	miR-34a plays a role in cardiac aging, and cardiac cell damage	miR-34a increases in the heart and results in cell death and fibrosis following acute myocardial infarction. Decreasing production could decrease severity of MI.	Boon et al. (2013)
Alzheimer’s	miR-34a regulates inflammation and plaque formation in individuals with Alzheimer’s	By modulating M1//M2 macrophage pathway miR-34a regulates inflammation which could contribute to the understanding of the currently unknown role of inflammation has on Alzheimer’s.	Sarkar et al. (2019)
Osteoporosis	miR-34a blocks osteoporosis and bone breakdown	By inhibiting osteoclast formation as well as interrupting the TGIF2 pathway, increasing levels of miR-34a is beneficial in the slowing of progression of osteoporosis	Nobrega et al. (2020)

Since the beginning of this decade, researchers have been examining the function that miRNAs play in stroke, and they have revealed that several different miRNAs either increased or decreased in response to stroke (Sonoda et al., 2019). Researchers have even found that serum miRNA levels may be used to predict the risk of cerebrovascular disease prior to the occurrence of stroke (Sonoda et al., 2019; Kadir et al., 2022). Some of these includes the regulation of neurogenesis (miR-18a; −19a; −124), inflammation (miR-26; 34a; −145; −424a), post-stroke angiogenesis (miR-15a; −210), oxidative stress (miR-145; −424), apoptosis (miR-181a; −592), BBB disruption (miR-155; −320; −34a), synaptic plasticity (miR-34a; −134; −193a; −326) and excitotoxicity (miR-223) (Bansal et al., 2019). Finding an association between specific miRNA molecules and ischemia events is a common objective for numerous labs studying these molecules in basic science, with the ultimate aim of developing a pharmacological compound that has the potential to inhibit the production of miRNA. Despite the fact that research is moving in the direction of employing these molecules as diagnostic and therapeutic markers for disease, more work has to be done to elucidate the complex processes behind the pathophysiology of stroke, and the even more convoluted role miRNA has in the disease process.

## 3. Association of miR-34a with ischemic stroke

Previously, it had been determined that miR-34a functions as a tumor suppressor gene by regulating cell expression via a positive feedback loop with p53 (Yamakuchi et al., 2008). The miR-34a gene has been abundantly found in the brain and is transcribed from chromosome 1p36, which is involved in apoptosis, differentiation, development, and cell cycle arrest (Lodygin et al., 2008). Numerous studies have identified a post-stroke increase in miR-34a levels, which may also play a role in the mitochondrial dysfunction seen in the stroke patients (Liang and Lou, 2016; Ren et al., 2019). Furthermore, increased levels of miR-34a have also been demonstrated to make the BBB more permeable by reducing blood cytochrome c (CYC) levels (Bukeirat et al., 2016; Hone et al., 2020). Decreased levels of cytochrome care additionally associated with mitochondrial dysfunction and an increase in reactive oxygen species (ROS) in the area around the stroke. Fan et al. demonstrated that higher levels of miR-34a were associated with lower expression of three electron transport chain (ETC) genes, NDUFS8, COX5b, and ATP5a1 (Fan et al., 2017). These findings suggest increased miR-34a disrupts the ETC which results in increased formation of reactive oxygen species (ROS), further contributing to oxidative damage.

Antagonism of miR-34a using gentamycin in an established *in vitro* stroke cell line showed increased cell viability and protected cells against oxidative damage (Izadi et al., 2023). Increased miR-34a has also been correlated with a decrease in brain derived neurotrophic factor (BDNF) (Zhu et al., 2021). BDNF has been shown to improve the ability of the brain to repair after a stroke (Liu et al., 2020). The inverse relationship that miR-34a has with BDNF contributes to the finding that miR-34a increases post-stroke are deleterious to the healing process (Abdolahi et al., 2022). MiR-34a also interacts with NF-κB, STAT1, and CREB transcription factors, all of which have previously been correlated positively with inflammation (Li et al., 2012; Sarkar et al., 2016; Ren et al., 2019). It has been shown that miR-34a also induces complement activation, disrupts innate immune signaling and dysregulates the expression of several key phosphoproteins involved in neurotropism and synaptic signaling (Chua and Tang, 2019; Mancha et al., 2020). MiR-34a knockdown was shown to reduce infarct size, and alleviated neurons in mice that were exposed to cerebral ischemia reperfusion (Wang et al., 2019), suggesting that miR-34a plays a negative role post ischemia reperfusion process. MiR-34a may exert its effects by controlling the regulation of myeloblastosis transcription factor (MYB) gene that is activated by apoptotic stimuli (Zauli et al., 2011; Choi et al., 2016). More research is warranted to explore the exact mechanism since marked increase in apoptosis near cerebral vascular events including stroke is a hallmark of the disease. Levels of miRNA-34a were found to be elevated in acute ischemic stroke patient samples and a negative association was found with miRNA-34a levels and NIHSS scores (Liang and Lou, 2016). A growing body of evidence also points to antagonism of miR-34a levels *in vivo* as a factor to improve stroke outcomes. The studies focus on miR-34a in disease models have been summarized in Table 2. In summary, miRNA-34a has a wide range of diverse effects that all contribute to the pathogenesis of stroke. Although, there are various proposed theories, as discussed above, it seems evident that there are still certain components in the pathway between miR-34a and stroke that remains to be clarified. Further solidifying the importance of the role miR-34a has in stroke.

**Table 2:** Role of miR-34a in stroke pathogenesis.

Experimental model	Expression levels of miR-34a	Study type	Mechanism	Significance	References
Cerebrovascular endothelial cells (CECs)	Increased	*In-vitro*	Increased BBB permeability. Disrupted tight junction protein ZO-1 Decreased mitochondrial oxidative phosphorylation Reduced ATP production Reduced ATP production Decreased cytochrome c levels	- Foundational study for the role of miR-34a in stroke pathogenesis.	Bukeirat et al. (2016)
Rat model (MCAO)	Increased	*in vivo*	Brain tissue injury Decreased Nissl’s bodies Increased infarction level Increased apoptosis Decreased platelet-derived neurotrophic factor (PDNF) Decreased Notch1/hypoxia-inducible factor-1α (HIF-1α) signaling pathway	miR-34a knockdown could alleviate brain tissue injury and neuronal apoptosis by activating the Notch1/HIF-1α signaling pathway	Wang et al. (2019)
Murine model (tMCAO)	Increased	*In-vivo* *In- vitro*	Increased miR-34a levels in pCECs at the time of BBB opening Knockout of miR-34a Reduced BBB permeability Alleviated tight junction disruption Improved stroke outcomes Purified pCECs from miR34a^−/−^ shows interaction with cytochrome c	Knockout of miR-34a improved BBB disruption and stroke outcomes.	Hone et al., (2020)
Rat PC12 cells with miR-34a antagonist (Gentamycin)	Decreased	*in vitro*	Decreased levels of miR-34a, Increased levels of caspase 3, SIRT1 p53. Reduced apoptosis	- Gentamycin can inhibit miRNA biogenesis pathway - Gentamycin enhanced cell viability and ameliorated cell death in PC12 model of stroke	Izadi et al. (2023)
Human ischemic stroke patients Rat model (MCAO)	Increased	Clinical*in-vivo*	Increased expression of miRNA-34a-5p in plasma Negative correlation between miRNA-34a-5p and NIHSS scores and infarct volume Increased expression of miR-34a both in blood and brain	Potential regulatory role of miRNA-34a-5p in acute ischemic stroke	Liang and Lou (2016)

### 3.1. miR-34a increases BBB permeability

Despite multiple circulating theories on the mechanism of miR-34a to worsen stroke outcomes, one of the most supported is its interaction with the BBB. The BBB maintains brain homeostasis by providing a tight, highly controlled barrier of entry via cerebrovascular endothelial cells (CECs). This provides the brain immune privilege of the CNS, sequestering it off from the rest of the body’s circulation (Banks, 2015). Stroke disrupts this barrier and increases vascular permeability in a two-step- fashion. First, in early reperfusion, endothelial transcytosis is the leading factor, and in the second phase, tight endothelial junctions start to give way (Bernardo-Castro et al., 2020; Dong et al., 2020). Tight junction protein complexes are dynamic complexes that can change in response to events such as stroke (Abdullahi et al., 2018). The tight junctions are made up of occludin and claudin proteins as well as adhesion junctions and accessory proteins. Tight junctions are regulated physiologically by protein modification, relocation, and degradation (Jiang et al., 2018).

Stroke-related studies have described that BBB opens at 6 h post-tMCAO and closed at 24 h (Hone et al., 2018), while miR-34a levels also shown significantly elevated in brain ischemic hemispheres at 6 h and 24 h post-stroke reperfusion (Ren et al., 2019). This shows strong association of miR-34a with BBB permeability post- stroke. This phenomenon was further verified by assessing miR-34a levels in purified pCECs of experimental stroke mice. Surprisingly, elevated miR-34a was most prominent in pCECs from ipsilateral hemispheres at 6 h post-tMCAO, consistent with the BBB opening time point (Hone et al., 2020). This data reveals the potentially important regulatory effects of miR-34a in BBB disruption during the early phase of stroke reperfusion.

Another study using *in vivo* miR-34a^−/−^ mice model suggest that the increases in levels of miR-34a increases the BBB permeability by decreasing expression of occludin, claudin-5 and zonula occludens-1 (Hone et al., 2020). Whereas overexpression of miR-34a increases BBB permeability and compromises BBB tight junctions (Bukeirat et al., 2016; Sarvari et al., 2020). However, bioinformatic analysis suggest that none of previously mentioned tight junctions are the direct targets of miR-34a. The levels of miR-34a increase most significantly 6 h after stroke which corresponds with the well documented BBB opening time of 6 h that has been noted across the literature on stroke (Merali et al., 2017). Despite the finding that tight junction permeability increases with increased levels of miR-34a the exact mechanism is yet to be revealed.

**Figure 2:**
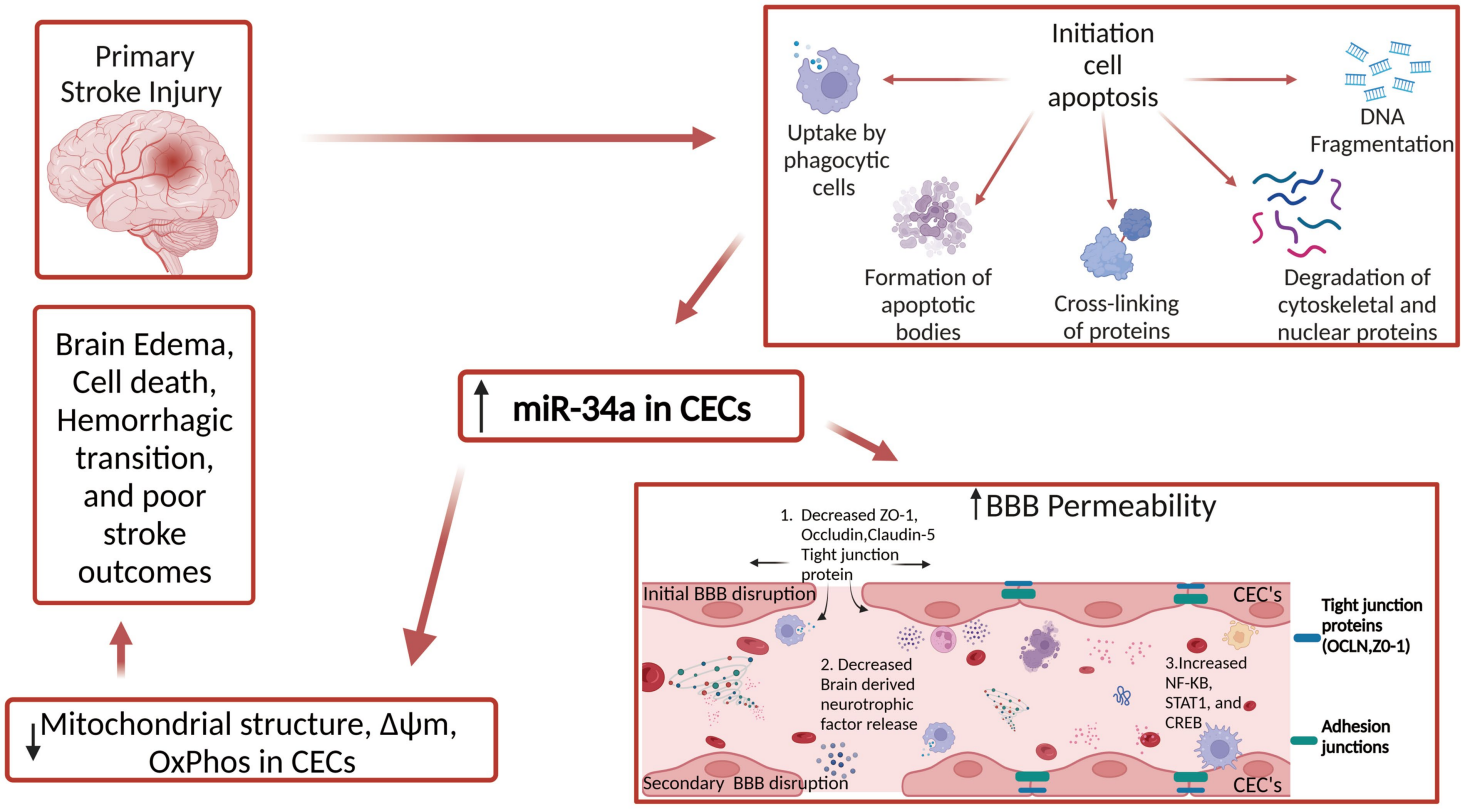
Role of miR-34a in BBB permeability in stroke. Mir-34a decreases stability of several tight junction proteins through unknown mechanism. This results in increased pro-inflammatory cytokines and other unwanted molecules entering the brain. Mir-34a also results in increase of several transcription factors that produce inflammatory cytokines. Lastly, brain derived neurotrophic factor (BDNF) levels decrease in response to MIR-34a level increase post stroke which contributes to weakened BBB function.

Multiple investigations have demonstrated that reduced intracellular calcium downregulates mitochondrial metabolism, which in turn results in decreased mitochondrial function (Song et al., 2022). Additionally, it has also been shown that increased intracellular calcium is one of the primary early events in ischemic stroke (Chung et al., 2015). During ischemic stroke, cells experience intracellular calcium overload due to depletion of ATP and subsequent ion pump dysfunction. BBB permeability increase has been seen with increased tight junction malfunction (Stuart et al., 1996), as well as decreased calcium levels causing changes in cellular localization of occluding and zonula occluden actin binding (Ye et al., 1999). Both findings indicate that preserving a healthy calcium concentration balance is crucial for maintaining the integrity of BBB. Figure 2 shows the current understanding of the role miR-34a has in BBB permeability post-stroke. MiR-34a appears to also operate as a regulator of store-operated calcium entry (SOCE) and calcineurin signaling. Furthermore, increased levels of miR-34a caused a depletion of endoplasmic reticulum calcium content and a decreased calcium influx through calcium mediated release calcium channels (Diener et al., 2018), however, it remains to be determined if miR-34a regulates calcium transport pathway in stroke. The knowledge of miR-34a and its involvement in stroke stops at the interaction the molecule has with the BBB more specifically endothelial cells and tight junctions. As BBB is also composed by astroglia, pericytes, and junctional complexes Therefore, more in-depth research is needed to elucidate miR-34a interaction with other cells. This will provide a more conclusive understanding of the role miR-34a has with stroke.

Brain capillary endothelial cells have a mitochondrial density that is two times denser than systemic capillaries (Banks, 2015). This high mitochondrial density supplies the brain’s high energy demand, which consumes around 20% of the total oxygen consumption in the body at any point in time (Fantin et al., 2010). Cytochrome c (CYC) is a small heme protein that is found on the inner membrane of mitochondria whose function is to transfer electrons between the third and fourth enzyme complexes for energy generation. CYC also play a crucial role in apoptosis and when a cell receives an apoptotic stimulus, it is released into the cytosol. It allosterically activates apoptosis-protease activating factor 1 which is required for the maturation of caspase-9 and caspase-3 (Benchoua et al., 2001; Jemmerson et al., 2005). These caspase proteins both have been identified as key molecules in modulation of ischemic stroke. The reports from our group showed that miR-34a not only increases the BBB permeability but also reduces the mitochondrial functions by targeting CYC and negatively impacts the ischemic stroke outcome (Bukeirat et al., 2016). *In vitro* study shows that overexpression of increasing concentration of miR-34a correlates with reduction of mitochondrial respiration and level of ATP production in CECs (Bukeirat et al., 2016). Due to the double effect that CYC has in oxidative phosphorylation and apoptosis we believe that miR-34a mediated decrease of CYC is a crucial step in the progression of ischemic stroke. Therefore, reducing the amount of miR-34a and subsequent BBB damage would be a significant milestone in the treatment of stroke.

It is believed that miR-34a has a plethora of oxidative phosphorylation and glycolysis genes as its target. Some of the oxidative phosphorylation genes involved with miR-34a produce proteins such as NDUFC2, SDHC, and COX 10. Examples of glycolysis genes involved produce proteins such as H6PD, PFK1, and LDH. These target genes are components of the electron transport chain, and miR-34a acts to decrease protein expression, which has been correlated with severely reduced mitochondrial function (Sarkar et al., 2016). In conclusion, increased levels of miR-34a have been reported post ischemic stroke, and the current hypothesis explaining this increase point toward decreased mitochondrial function and therefore increased BBB permeability that stems from this increase in miR-34a. The exact molecular pathway for this decreased mitochondrial function is not known completely, however, a large list of target genes has been identified, and therefore more work needs to be done to establish the exact mechanism in which miR-34a works by.

### 3.2. Factors affecting miR-34a levels

While not directly correlated with the disease process of stroke, in individuals that suffer from hypertension, miR-34a also was significantly upregulated due to its target of TIGF2 in vascular endothelial cells. This is still significant when looking at the function of miR-34a because hypertension is a common precursor to stroke. Another factor to look at when discussing miRNA is the effect aging has on the amount of molecule in the body. Research has shown that levels of miRNA change as an individual ages (Noren Hooten et al., 2013). As for miR-34a it has been hypothesized that age dependent changes could have an impact on one’s outcome for stroke, however, research has yet to be done on the age-related increase in stroke. Studies have shown that the levels of miR-34a increase in the kidney as one ages (Bai et al., 2011), and that levels of miR-34a also increase in individuals with Alzheimer’s (Sarkar et al., 2019). Despite this trend between the two due to the variability of function miRNA has in the body in different cell types and regions of the body, no conclusions can be made on the factor age plays in miR-34a levels, but nonetheless gives researchers a sense of what to look for going forward.

## 4. miR-34a as a progressive and diagnostic biomarker for ischemic stroke

Traditionally, the diagnosis of stroke mainly depended upon examination by a clinical care provider, and various neuron-imaging techniques (Kisialiou et al., 2012; Whiteley et al., 2012). However, these diagnostic methods are only able to confirm the disease status of patients and do not serve to predict disease. Therefore, there is great need for a reliable and easily detectable circulating biomarker for acute ischemic stroke risk and/or outcome prediction.

With few exceptions, the miRNAs assessed to date increase after stroke. Whether the post-stroke increase in miRNAs is detrimental or beneficial is open to debate. Analyses of the interaction between ischemic pathology and miRNAs has yielded valuable insights into the brain injury progression and toward the development of biomarker panels and novel therapeutic targets. MiR-34a is of great interest because it has essential role in regulating mitochondrial function (Duarte et al., 2014). In neurons, miR-34a targets and reduces numerous glycolysis and OxPhos proteins (Sarkar et al., 2016); whereas in CECs, a more selective suppression of CYC C is seen, but is associated with substantial OxPhos reductions (Bukeirat et al., 2016). Patient-based studies have already reported some alterations in circulatory miR-34a-5p expression was increased in patients that persist for several weeks to months beyond the acute recovery period (Liang and Lou, 2016). The changing expression levels in blood samples was also correlated with brain samples obtained from rat models, which demonstrates the ability of miR-34a-5p as a potential biomarker for acute ischemic stroke (Liang and Lou, 2016). Similarly, we also observed circulatory increases in miR-34a expression in the blood of stroke patients and both plasma and CSF of experimental model of stroke, that further worsen stroke associated preclinical outcomes (Ren et al., 2019). *In vitro* study suggested that these changes may be driven at least in part by disruptions to blood brain barrier integrity and mitochondrial oxidative phosphorylation in endothelial cells. However, methodical genetic manipulation of miR-34a expression substantially impacted stroke-associated preclinical outcomes and lay the groundwork for future investigation of miR-34a as a progressive and diagnostic biomarker and a novel therapeutic target for ischemic stroke.

## 5. Perspectives and conclusion

The aforementioned research articles have demonstrated a positive correlation between elevated levels of miR-34a and contribution to the development of adverse effects associated with ischemic stroke. These findings strongly indicate that miR-34a not only diminishes expression of multiple tight junction proteins but also compromises the integrity of the BBB, thereby resulting in impaired mitochondrial energy metabolism and cell death. Collectively, these mechanisms exert negative regulation on the outcomes of ischemic events, such as stroke. Stroke affects over fifteen million individuals aged 65 and above annually. This review firstly dove into the biological processes governing miRNA generation and explores the significant impact these molecules exert on the pathogenesis of numerous diseases including stroke. Subsequently, the pertinent data concerning the role of miR-34a in stroke and various proposed mechanisms underlying its involvement were discussed. This section was followed by an in-depth analysis of the specific relationship between miR-34a and BBB permeability, lastly, factors influencing levels of miR-34a were examined.

Due to the substantial surge of research dedicated to miRNAs in recent years, there has been an exponential increase in our understanding of the underlying molecular mechanisms governing the diverse functions of thousands of different miRNAs. These functions are not only unique to each miRNA but are also intricately intertwined with the specific organs or tissues in which these miRNAs are expressed throughout the human body, with a single miRNA potentially having completely different functions in different scenarios. The complexity inherent to these molecules poses a formidable challenge in attaining a comprehensive understanding, sufficient for the development of clinically useful diagnostic tools and therapeutic interventions. Despite these barriers, miR-34a has a robust background of foundational basic science information to continue building upon. Although miR-34a levels are consistently associated with certain brain pathology and has been verified in cellular and animal disease models, it is yet unclear if an assessment of its levels in human samples could become a part of a profiling panel that would serve diagnostic and/ or prognostic purposes in neurological disorders, particularly stroke. MiR-34a’s usefulness in this regard should improve with further testing and analyses, particularly when probed in conjunction with a panel of other miRNAs. Additionally, the intimate role of miR-34a in the BBB function, presents an exciting avenue for therapeutic development. Coupled with advancements in nanoparticle-based pharmaceutical delivery methods, there emerges a potential solution to one of the longstanding issues plaguing clinical stroke treatment. These issues include a litany of clinical trials that showed no clinical beneficence, despite large amounts of basic science research suggesting beneficence of such treatments. Many of which were primarily rooted in the ineffective delivery of medications across the BBB. Similarly, miR-34a-based therapy also need efficient methods of delivery that bypasses the BBB. Furthermore, as we discussed earlier that the knockout of miR-34a improve stroke outcomes, suppression or inhibition of miR-34a might be crucial in the treatment of stroke. However, introducing miRNA antagomirs into the CNS is the main challenge in this field, suggesting that much more remains to be investigated.

In conclusion, the findings presented in this review greatly contribute to the knowledge base for researchers interested in further understanding of miR-34a. While the role of miR-34a in compromising BBB integrity and exacerbating the adverse effects of stroke is becoming clearer, there remains a need to delve deeper into the precise mechanisms that govern its action. Additionally, exploring the potential cross-talk between miR-34a and other molecular players involved in stroke pathogenesis could unveil novel therapeutic targets. Incorporating advanced techniques like single-cell sequencing and gene editing may provide a more comprehensive understanding of miR-34a’s influence on cellular responses post-stroke. Furthermore, assessing the temporal dynamics of miR-34a expression and its correlation with different stages of stroke progression could offer valuable insights into its potential as a prognostic biomarker.

## Author contributions

CP: Writing – original draft. ST: Writing – review & editing. SW: Writing – review & editing. HH: Writing – review & editing. AG: Writing – review & editing. HC: Writing – review & editing. XR: Conceptualization, Funding acquisition, Investigation, Supervision, Writing – review & editing. HC also has a role in funding acquisition.
